# Empowering women can improve child dietary diversity in Ethiopia

**DOI:** 10.1111/mcn.13285

**Published:** 2021-11-04

**Authors:** Kaleab Baye, Arnaud Laillou, Stanley Chitekwe

**Affiliations:** ^1^ Center for Food Science and Nutrition, College of Natural and Computational Sciences Addis Ababa University Addis Ababa Ethiopia; ^2^ Research Center for Inclusive Development in Africa (RIDA) Addis Ababa Ethiopia; ^3^ Nutrition Section UNICEF Ethiopia Addis Ababa Ethiopia

**Keywords:** complementary feeding, diet quality, dietary diversity, women empowerment

## Abstract

Women empowerment is an underlying factor of child feeding and nutrition. However, the lack of standardized measurements has made it difficult to design interventions that embed women empowerment and measure their impacts. This study aimed to assess temporal trends in women empowerment in Ethiopia and evaluate their contribution towards improving dietary diversity in infants and young children. We used women and child data from the Ethiopian Demographic and Health Survey 2005, 2011, and 2016, yielding a total sample of 6113 mother–child pairs. The survey‐based women's empowerment index (SWPER) developed and validated for use in Africa was used to assess three dimensions of women empowerment: (i) social autonomy, (ii) decision making; and (iii) attitude to violence. We used multiple‐linear and multivariable logistic regression to assess the associations between SWPER and the number of food groups consumed/and the minimum dietary diversity (MDD). To determine drivers of changes over time, a regression decomposition analysis was run. Women empowerment indices have improved over the 2005–2016 period, but a significant proportion of women had low standardized SWPER scores for autonomy/social independence (47%) and attitude to violence (49%) domains in 2016. SWPER autonomy and SWPER decision‐making scores were strongly associated with the odds of meeting MDD. Changes in women empowerment accounted for 17% of the improvements in MDD between 2005 and 2016. SWPER was a stronger predictor of the change in MDD, than known predictors like wealth, child age, and urban residence. As a critical underlying driver of child nutrition, women empowerment should be boldly addressed and integrated in nutrition interventions.

**Key messages**

- Women empowerment has improved between 2005 and 2016, but remained low and varies by region, rural–urban residence, and wealth quintile.
- Women empowerment accounted for 17% of the improvements in dietary diversity.
- SWPER was a stronger predictor of the change in dietary diversity, than known predictors like wealth, child age, and urban residence.
- Women empowerment should be boldly addressed and integrated in nutrition interventions.

## 1 INTRODUCTION

Despite some improvement, undernutrition remains a serious public health concern in low‐income countries (Victora et al., 2021). In 2019, 144 million children 6–59 months of age were stunted, and another 47.0 million were wasted (WHO, 2020), leading to dire economic and health consequences (Dewey & Begum, 2011; Laillou et al., 2020). Stunting reaches a peak during the period of complementary feeding (6–23 months of age), suggesting that inappropriate complementary feeding is a significant contributor to the burden of child malnutrition (Victora et al., 2010). This, along with the realization of the short and long‐term consequences of child undernutrition, led to the consideration of the first 1000 days, from conception to the child's second birthday, as a critical window of opportunity for the prevention of malnutrition (Baye & Faber, 2015).

A number of evidence‐based nutrition‐specific and nutrition‐sensitive interventions have been implemented, but progress has been too slow (Bhutta et al, 2013; Victora et al., 2021). Diet and diseases are the immediate causes of child undernutrition, while inadequate access to food, health, care and feeding practices are the underlying causes (UNICEF, 1990). With less than a fifth of children 6–23 months of age meeting the minimum dietary diversity (MDD) in Sub‐Saharan Africa (Baye & Kennedy, 2020), inadequate diet quality is a critical concern. In Ethiopia, the proportion of children that meet the MDD has increased, but only 12.5% of children met the MDD in 2016 (Baye, 2021; CSA & ICF., 2016). A growing number of studies have highlighted that the quality of nutrition education delivered, the unaffordability of nutrient‐dense foods, and the overall inequalities in access to economic and health services are critical underpinnings that are holding‐back progress (Abebe et al., 2016; Bachewe et al., 2017; Baye et al., 2020). However, little is known about how gender inequality or women empowerment influences this dynamics.

Women empowerment is a goal that in itself can promote good nutrition and health for both the women and the child, improve child care, as well as reduce poverty (Duflo, 2012; Meinzen‐Dick et al., 2019; Santoso et al., 2019). Consequently, understanding and measuring women empowerment is critical to contribute towards the achievement of the Sustainable Development Goal (SDG) 5 “achieve gender equality and empower all women and girls”, but also SDG2 “ending all forms of malnutrition” and other related SDGs (Baye, 2017). Unfortunately, the lack of standardized measurements of women empowerment has made it difficult to monitor and design interventions that embed women empowerment (Ewerling et al., 2017). Although efforts were made to develop indicators that capture gender inequality, most relied on national‐level aggregate data, limiting their use for within country comparisons (Ewerling et al., 2021).

Using various women empowerment definition and measures, studies have shown that women empowerment is associated with better child nutrition. Unfortunately, these measures of women empowerment are not always comparable and are not always systematically captured in national surveys; hence, making time‐trend analyses difficult. More recently, a survey‐based women's empowerment (SWPER) index was developed and validated for use in Africa (Ewerling et al., 2017). The SWPER is an individual‐level indicator that allows time‐trend analyses and between country comparisons. The SWPER captures three women empowerment dimensions: (i) attitude to violence, (ii) decision making, and (iii) social autonomy/independence. Using the 2016 Ethiopian Demographic and Health Survey (DHS), Mekonnen et al. (2021) showed that the SWPER index is associated with reduced odds of child growth faltering. However, how the SWPER index (i.e., women empowerment) has changed over time, and how it is related to child dietary diversity remains unknown. All the three dimensions captured by the SWPER could potentially influence child feeding, but this has not been evaluated in the context of Ethiopia, where child and maternal nutrition remains a public health concern. Therefore, this study aimed to assess trends in women empowerment and evaluate their contribution towards improved infants and young child feeding.

## 2 METHODS

### 2.1 Overview and data source

We pooled data from the last three rounds of the Demographic and Health Surveys (DHS 2005, 2011, 2016). The DHS is a nationally and regionally representative survey that uses a stratified cluster sampling design with households drawn randomly at the last stage. We evaluated the association between women empowerment, measured using SWPER, and dietary diversity and the proportion of children that meet the MDD. We restricted our analyses to married/partnered women with children 6–23 months of age, as the SWPER index only considers partnered/married women.

### 2.2 Outcome variables

The primary outcome of interest was the updated MDD, which includes breastmilk as one food group. The MDD is met when at least five of the eight food groups are consumed in the 24 h prior the survey (Baye & Kennedy, 2020). The eight food groups used to construct the MDD are (i) breastmilk; (ii) grains, roots and tubers; (iii) legumes and nuts; (iv) dairy products; (v) flesh foods (meat, fish and poultry); (vi) eggs; (vii) vitamin A‐rich fruits and vegetables; and (viii) other fruits and vegetables.

### 2.3 Explanatory variables

Women empowerment was quantified using the recent SWPER index developed and validated for use in Africa (Ewerling et al., 2017). In comparison with existing women empowerment index, the SWPER index has been tested (i) in far more countries in Africa than other alternative indicators (Ewerling et al., 2017); (ii) has more recently been validated using data from all low and middle income countries. Besides, the index was found to be consistently associated with child development (Ewerling et al., 2020) and coverage of reproductive, maternal, newborn and child health interventions (Ewerling et al., 2021). Both child development and access to health interventions are associated with child diet quality and nutritional outcomes (Baye et al., 2020; Gould, 2017); hence, making the SWPER index more relevant to our study.

SWPER scores for (i) attitude towards violence, (ii) social independence (autonomy) and (iii) decision‐making domains of empowerment were calculated. The indices are generated based on the DHS empowerment module that assesses women's involvement in household decisions, employment and type of earnings, control of resources, opinion on partner violence, and ownership of house/land. The details related to the construction of the SWPER are presented in the Table S1. The score was then standardized using the mean and standard deviation for the Sub‐Saharan African region, as described in detail elsewhere (Ewerling et al., 2020). A score of zero represented the average value for the African region, and the following cut‐offs were used to categorize SWPER index into low, medium, and high SWPER scores:

- Attitude to violence: low: ≤ −0.7; medium: −0.7 to 0.4; and high: >0.4
- Social independence: low: ≤ −0.559; medium: −0.559 to 0.293; and high: >0.293
- Decision‐making: low: ≤ −1.0; medium: −1.0 to 0.6; and high: >0.6

As a measure of household socioeconomic status, we computed a household wealth index scores based on 32 common assets and services using principle component analyses (PCA). The constructed wealth index, unlike the relative wealth index constructed by the DHS, allows an absolute measure of wealth that is comparable over time (Rutstein & Staveteig, 2014). The comparative wealth index (CWI) was constructed for both urban and rural, based on common assets and services that are available across surveys. This allowed the CWI to become an absolute measure of wealth that can be compared over time (Rutstein & Staveteig, 2014).

Maternal and child characteristics such as age of the child (in months), gender, antenatal care (ANC) visits during pregnancy, and the number of children (<5 years of age) in the households were captured. Rural–urban residence and administrative regions were included as control variables.

### 2.4 Statistical analyses

To assess associations between SWPER and the number of food groups consumed and MDD, we run multiple linear regression and multivariable logistic regressions, respectively. First a bivariate model was run, and all variables with a *p* value <0.2 were included in the multivariate model and multivariable logistic regression. Multivariate regression model was run with the number of food groups as outcome, whereas multivariate logistic regression was run with MDD as outcome variable. In both models, the following covariates were included in the model: (i) SWPER: attitude to violence, (ii) SWPER: autonomy/social independence, (iii) SWPER: decision making SWPER score, (iv) mother/caregiver age in years, (v) residence, (vi) wealth score (0–10), (vii) attended 4+ ANC visits, (viii) number of children under five in the household, (ix) child age in months and (x) sex of a child. To control for non‐time variant (region) and other time‐variant unmeasured variables, we have controlled for year of survey.

The multiple linear regression model for the number of food groups (*Y*) a child *i* ate at time *t* and vectors of SWPER index (*X*), other control variables (*Z*), a vector of year dummy variables (*T*) and a standard error term (*ε*_*i*,*t*_) is represented as follows:

(1) $$Y_{i,t}=\beta X_{i,t}+\beta Z_{i,t}+T+\varepsilon_{i,t}$$

Our multivariable logistic regression model for MDD (*Y* = 1: a child receive foods from five or more food groups) of a child *i* at time *t* and vectors of SWPER index (*X*), other control variables (*Z*), a vector of year dummy variables (*T*), and a standard error term (*ε*_*i*,*t*_) is represented as

(2) $$\frac{p\left(Y_{i,t}=1\right)}{1-p\left(Y_{i,t}=1\right)}=\beta X_{i,t}+\beta Z_{i,t}+T+\varepsilon_{i,t}$$

To determine drivers of changes in MDD over time, a regression decomposition analysis was run. However, given that linear decomposition or standard Blinder–Oaxaca (BO) decomposition for binary dependent variables provides misleading estimates due to path‐dependency‐related issues (Fairlie, 2017), we used a nonlinear decomposition technique for the logistic model. To determine the contribution of women's empowerment and other covariates on the predicted changes in MDD over time, a Fairlie's nonlinear decomposition was run. Additionally, to accommodate the sampling nature of DHS, the decomposition analyses used sampling weights. The nonlinear decomposition equation of, $Y=F\left(X\hat{\beta }\right)$, can be expressed as

(3) $$\begin{array}{c} {\bar{Y}}^{2016}-{\bar{Y}}^{2005}=\left(\sum_{i=1}^{N^{2016}}\frac{F\left(X_{i}^{2016}{\hat{\beta }}^{2005}\right)}{N^{2016}}-\sum_{i=1}^{N^{2005}}\frac{F\left(X_{i}^{2000}{\hat{\beta }}^{2005}\right)}{N^{2005}}\right)\\ +\left(\sum_{i=1}^{N^{2016}}\frac{F\left(X_{i}^{2016}{\hat{\beta }}^{2016}\right)}{N^{2016}}-\sum_{i=1}^{N^{2016}}\frac{F\left(X_{i}^{2016}{\hat{\beta }}^{2005}\right)}{N^{2016}}\right) \end{array}$$

where $N^{j}$ denotes the sample size of each time points (*j* = 2000, 2016), $F\left(\right)$ is the cumulative distribution function of the logistic distribution, ${\bar{X}}^{j}$ and ${\hat{\beta }}^{j}$ are the vectors of each time points means of determinants factor and the vector of estimated coefficients, respectively.

Data management and statistical analysis were conducted in Stata Version 16.1.

## 3 RESULTS

All the mean SWPER scores increased significantly over the last three rounds of DHS (Table 1), moving the mean scores from a low to a medium category during the 2005–2016 period. However, disparities in women empowerment scores were observed by rural–urban residence and wealth quintile. Women in urban areas had significantly higher SWPER scores than those in rural areas (*p* < 0.01). Similarly, women from the richest households (fifth and the fourth wealth quintile) had a markedly higher SWPER score than those from households from the lower wealth quintiles (*p* < 0.01). Between 2005 and 2016, significant increases in the proportion of children that met the MDD, SWPER score, wealth score and proportion of women that attended 4+ ANC were noticed (Table S2). Despite these increases in SWPER scores, close to half of the women had a low attitude to violence and social independence/autonomy SWPER scores in 2016. Also noteworthy is the statistically significant regional variation in all the women empowerment domains (Table S3). In 2016, the Afar region had the lowest SWPER score, whereas Addis Ababa had the highest score.

**Table 1:** Trends in women empowerment (SWPER index) by rural–urban and wealth quintile

Characteristics	SWPER score: attitude to violence, mean (*SD*)			*p* value^a^	SWPER score: autonomy/social independence, mean (*SD*)			*p* value	SWPER score: decision making, mean (*SD*)			*p* value
	2005	2011	2016		2005	2011	2016		2005	2011	2016	
Residence												
Rural	−1.067 (0.973)	−0.855 (1.059)	−0.687 (1.117)	<0.01	−0.56 (0.622)	−0.515 (0.639)	−0.458 (0.694)	<0.01	−0.164 (0.766)	0.021 (0.737)	0.207 (0.783)	<0.01
Urban	−0.296 (1.014)	−0.076 (1.086)	0.18 (0.951)	<0.01	0.169 (1.024)	0.046 (0.992)	0.466 (1.135)	0.05	0.56 (0.747)	0.347 (0.756)	0.659 (0.692)	0.37
Wealth quintile												
Q1: Poorest	−1.12 (0.896)	−0.963 (1.02)	−0.714 (1.162)	<0.01	−0.5 (0.676)	−0.569 (0.611)	−0.535 (0.648)	<0.01	−0.291 (0.82)	−0.106 (0.78)	0.151 (0.812)	<0.01
Q2: Second	−1.093 (0.957)	−0.935 (1.033)	−0.706 (1.102)	<0.01	−0.621 (0.598)	−0.535 (0.61)	−0.529 (0.597)	<0.01	−0.185 (0.779)	−0.009 (0.734)	0.177 (0.795)	<0.01
Q3: Middle	−1.083 (1.012)	−0.776 (1.077)	−0.758 (1.097)	<0.01	−0.585 (0.611)	−0.524 (0.631)	−0.463 (0.687)	0.07	−0.071 (0.708)	0.054 (0.657)	0.252 (0.819)	<0.01
Q4: Fourth	−1.04 (1)	−0.734 (1.091)	−0.517 (1.079)	<0.01	−0.541 (0.633)	−0.445 (0.633)	−0.26 (0.826)	<0.01	−0.086 (0.749)	0.125 (0.722)	0.261 (0.729)	<0.01
Q5: Richest	−0.578 (1.064)	−0.177 (1.112)	−0.049 (1.09)	<0.01	−0.182 (0.883)	−0.014 (1.001)	0.242 (1.125)	<0.01	0.169 (0.838)	0.365 (0.76)	0.549 (0.688)	<0.01
National	−1.012 (0.996)	−0.754 (1.094)	−0.584 (1.133)	<0.01	−0.507 (0.686)	−0.442 (0.72)	−0.348 (0.817)	<0.01	−0.112 (0.788)	0.064 (0.748)	0.261 (0.787)	<0.01
Low (%)^b^	67.9	55.2	48.9		53	48.8	46.6		14.1	12.1	9.9	
Medium (%)	17.3	21.3	20.1		34.4	38	35.6		74	71.5	68.1	
High (%)	14.8	23.5	31.0		12.5	13.2	17.8		11.8	16.4	22	

Abbreviations: SWPER, survey‐based women's empowerment index.

^a^ *p* values from comparison of means across time using linear regression. Differences between mean scores within a given year (2005,11,16) was statistically significant by rural–urban (independent *t* test) and by wealth quintile (linear regression) at *p* < 0.01 (not shown).

^b^ Proportion of women with low, medium and high SWPER scores; *SD*, standard deviation.

**Figure 1:**
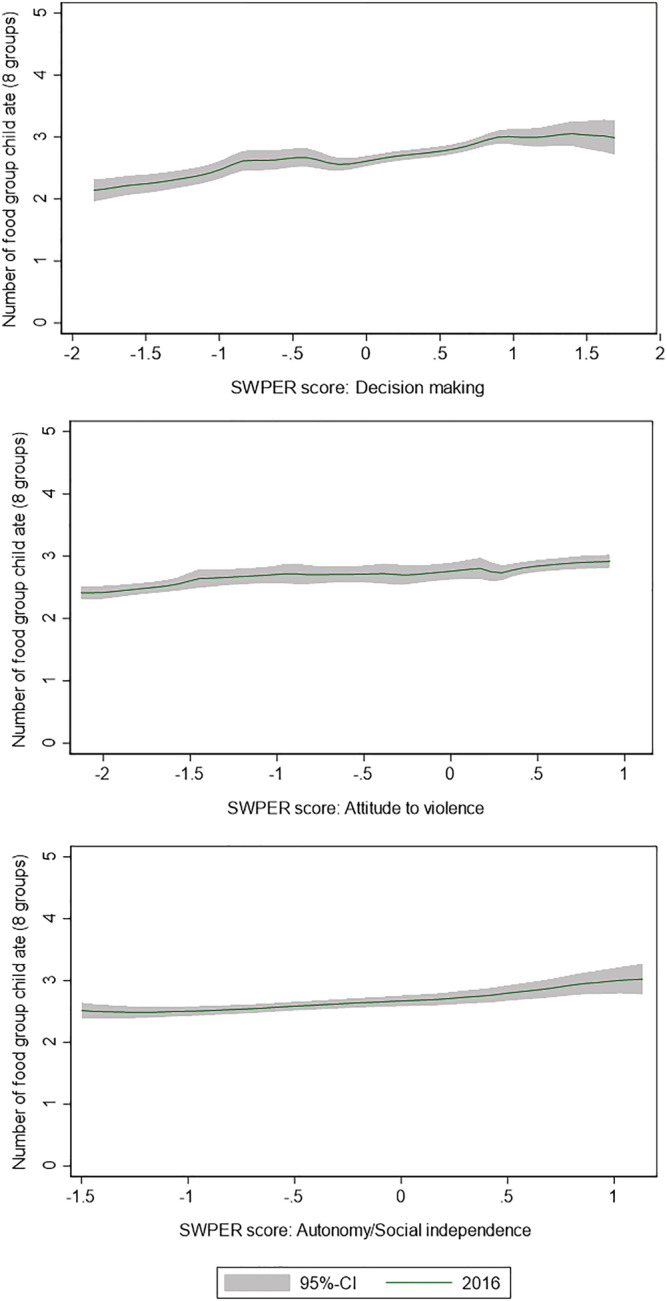
Relationship between food groups consumed and women empowerment

**Figure 2:**
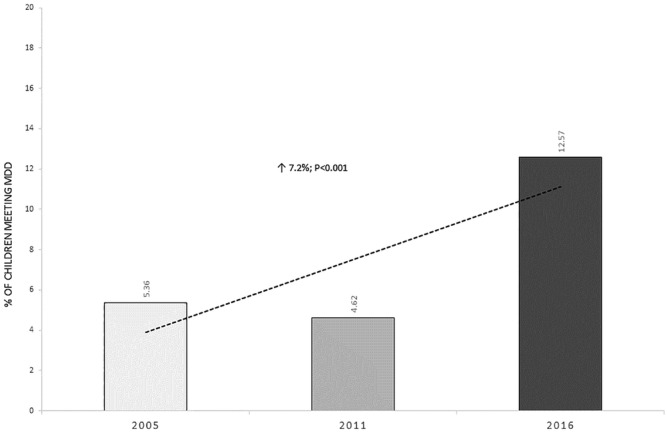
Proportion of children 6–23 months meeting the minimum dietary diversity score, 2005–2016

Figure 1 shows the relationship between SWPER score and the number of food groups consumed. The average number of food groups consumed increased linearly with increases in the SWPER scores. Similarly, the proportion of children that met the MDD increased from 2005 to 2016 (Figure 2); an increase significantly associated with the increases in SWPER scores, as confirmed by our regression models (Table 2). All the SWPER scores were significantly associated with the odds of meeting the MDD, but the SWPER score for attitude to violence was not associated with the consumption of additional food groups. The SWPER index remained a significant predictor of diet quality in children 6–23 months of age, even after accounting for known cofounders in the multivariable regression (Table 3). The SWPER decision‐making and autonomy domains were significant predictors of MDD. About 17% of the observed changes in MDD between 2005 and 2016 are explained by changes in the three SWPER scores.

**Table 2:** Determinants of number of food groups a child ate and MDD among children aged 6–23 months in pooled regression models of EDHS 2005, 2011 and 2016: (*n* = 6113)

Variables	Food groups (out of 8)^a^		MDD (5+ out of 8)^b^	
	β	[95% CI]	OR	[95% CI]
SWPER: attitude to violence	0.025	[−0.005, 0.055]	1.109*	[1.005, 1.223]
SWPER: autonomy/social independence	0.098**	[0.051, 0.146]	1.254**	[1.105, 1.424]
SWPER: decision‐making SWPER score	0.067**	[0.031, 0.104]	1.275**	[1.125, 1.445]
Respondent's current age in years	−0.003	[−0.008, 0.001]	0.974**	[0.957, 0.992]
Residence: urban	0.179*	[0.017, 0.342]	1.826**	[1.195, 2.790]
Wealth score (0–10)	0.117**	[0.082, 0.153]	1.157**	[1.058, 1.266]
Attended 4 + ANC visits	0.116*	[0.024, 0.208]	1.255	[0.974, 1.617]
Number of children 5 and under	−0.003	[−0.047, 0.041]	0.983	[0.836, 1.155]
Child age in months	0.052**	[0.046, 0.058]	1.068**	[1.049, 1.088]
Constant	1.929**	[1.707, 2.152]	0.027**	[0.012, 0.059]

Abbreviations: CI, confidence interval; EDHS, Ethiopian Demographic and Health Survey; MDD, minimum dietary diversity; OR, odds ratio; SWPER, survey‐based women's empowerment index.

^a^ Multiple linear regression: adjusted for region and survey year.

^b^ Multivariable logistic regressions: *adjusted for region and survey year*.

* *p* < 0.05 (regression model with a robust variance estimator).

** *p* < 0.01 (regression model with a robust variance estimator).

**Table 3:** Estimated contribution of each explanatory variable, in actual and percentage points for MDD among children aged 6–23 months between 2005 to 2016: Fairlie's nonlinear decomposition analysis (*n* = 3596)

Decomposition	MDD	Contribution (%)
DHS‐2016	0.126	—
DHS‐2005	0.054	—
Difference (2016–2005)	0.072	—
Total explained change	0.031	43
Total unexplained change	0.041	57

Explained part			
Variables	Contribution	95% CI	Contribution (%)
Region	0.008**	[0.006, 0.011]	11
SWPER: decision‐making SWPER score	0.007**	[0.003, 0.011]	10
SWPER: autonomy/social independence	0.004**	[0.002, 0.006]	6
SWPER: attitude to violence	0.001	[−0.003, 0.005]	1
Respondents' current age in years	0.001	[−0.000, 0.001]	1
Attended 4+ ANC visits	0.004*	[0.000, 0.007]	5
Residence: urban	0.003**	[0.002, 0.005]	5
Wealth score (0–10)	0.003	[−0.006, 0.011]	4
Child age in months	0.002**	[0.001, 0.003]	3
Number of children 5 and under	−0.001	[−0.003, 0.000]	−2

Abbreviations: ANC, antenatal care; CI, confidence interval; DHS, Demographic and Health Survey; MDD, minimum dietary diversity; SWPER, survey‐based women's empowerment index.

* *p* < 0.05.

** *p* < 0.01 (adjusted region).

## 4 DISCUSSION

To our knowledge, the present study has for the first time highlighted that women empowerment indices in Ethiopia have improved over the 2005–16 period, but also showed that the SWPER scores remained negative for autonomy/social independence and decision‐making domains. All the SWPER domains were associated with dietary diversity, but autonomy and decision‐making SWPER domains were more strongly associated with the odds of meeting MDD. Women empowerment, assessed by the SWPER index, was a stronger predictor of the change in MDD (2005–2016) than common predictors like wealth, child age, and urban residence.

The recognition of the first 1000 days as a window of opportunity to improve child nutrition has led to the identification and scale‐up of nutrition interventions, but only a few nutrition‐specific interventions like ANC and iron‐folic acid supplementation directly target women (Bhutta et al., 2013). Once the child is born, such nutrition interventions almost entirely shift their focus to the child, providing little attention to the underlying maternal factors that could determine the very success of the interventions delivered to the child. This is unfortunate given that the effectiveness of critical interventions like the promotion of breastfeeding, adequate complementary feeding, growth monitoring, can all rely on the caregivers' agency to adopt recommendations, which in‐turn depends on how empowered the mother is (Ewerling et al., 2021; Matare et al., 2021). Fortunately, in the past decade, some efforts were made to also promote nutrition‐sensitive interventions including those that aim to contribute to women empowerment.

In many African settings, grandparents, siblings, religious leaders and neighbours play a significant role in influencing infant and young child feeding, making it harder for the mother to go against their recommendation when found inaccurate or disadvantageous for her child (Karmacharya et al., 2017). However, women with higher self‐autonomy and decision‐making scores can be assertive enough to apply nutrition messages, even when they go against widely held beliefs. Indeed, both self‐autonomy and decision‐making SWPER scores were strongly associated with the odds of meeting the MDD, a finding that is in line with earlier studies (Amugsi et al., 2016; Ickes et al., 2018; Shroff et al., 2011).

Likewise, the SWPER attitude to violence score was associated with MDD, and earlier studies have also found attitude to violence to influence the use of modern contraceptives, women's mental health, child caring and early child development (Ewerling et al., 2017; Fenta et al., 2020; Pedroso et al., 2020). Exposure to domestic violence (toxic stress) can also create a vicious cycle of compromised child development, maternal depression and poor child caring and nurturing practices (Black et al., 2020). Indeed, a recent study from Ethiopia found that inadequate feeding practices and undernutrition were associated with maternal depression (Anato et al., 2020).

Although integrating women empowerment into nutrition interventions has the potential to lead to more effective programmes by improving access to health care and services (e.g., delivery in health facility), adoption of health and nutrition messages, and better provisioning of a nurturing and child caring environment; very few examples of such integrated interventions exist to date (Black & Kowalski, 2021; Matare et al., 2021). In the Ethiopian context, women empowerment through income‐generating activities like small‐scale food processing could be part of social protection programmes. For example, minimal processing of fruits (e.g., dehydration) in surplus producing areas like the Southern region of Ethiopia, can potentially extend the shelf‐life of fruits, reduce postharvest loss and enable year‐round fruit availability, but also more importantly contribute to improving complementary feeding. This, if supported by behavioural change communication that creates demand for healthier diets and nutrition services, can be more effective in improving diets equitably. Indeed, using a different measurement of women empowerment (i.e., Women's Empowerment in Agriculture Index [WEAI]), Malapit and Quisumbing (2015) showed that women empowerment was strongly associated with diet quality in Ghana. However, how best to integrate food, health and social protection‐systems would warrant more thorough investigation.

The present study has a number of limitations that need to be considered when interpreting our findings. First, women empowerment is a complex concept closely tied to cultural norms, which can be difficult to fully and accurately account for, but although imperfect, the SWPER index is the best available, validated, individual‐level index. Second, an inherent limitation of the SWPER index is the fact that it is limited to partnered/married women, is not comprehensive as it only relies on data captured by the DHS, and it also does not measure women empowerment as a process. Third, our decomposition analyses rely in explaining temporal changes in MDD and food groups consumed. This analysis will only identify factors whose changes were related to the changes in outcome, and hence tell nothing about important predictors of diet quality that have not or changed little during the period of 2005–2016. Fourth, although we pooled data from multiple time points, the study remains cross‐sectional and thus does not allow causal inferences to be made. Finally, the association of women empowerment with child feeding was assessed using MDD as a proxy and did not consider all aspects of child feeding, partly because the other indicators like minimum meal frequency are not captured in the 2005 DHS round.

Notwithstanding the above limitations, this study has for the first time evaluated the relationship between women empowerment and child feeding, using the recently validated SWPER index for Africa. Although much progress is still needed, the positive changes in women empowerment over the 2005–2016 period predicted a significant proportion of the changes in the proportion of children that met the MDD. Empowering women can present immense opportunities to improving maternal and child nutrition, but this opportunity has so far been largely overlooked. As a critical underlying driver of child nutrition, women empowerment should be more boldly addressed and integrated in nutrition interventions. Women empowerment can be an entry point to effectively integrate actions across food, health and social protection systems to improve complementary feeding in Ethiopia and other similar settings.

## ACKNOWLEDGMENT

This study was funded by UNICEF Ethiopia.

## CONTRIBUTIONS

K. B., A. L. and S. C. conceived the study; K. B. prepared and analysed the data with inputs from A. L.; K. B. wrote the first draft. All authors have read and approved the final manuscript.

## Supporting information


**Table S1:** Description of variables included in the construction of the SWPER index
Table S2: Trends and changes in means or percentage of child diet and child diet determinants among children aged 6–23 months in Ethiopia for 2005, 2011, and 2016.
Table S3 Change in SWPER index between 2005 and 16, by region

## Data Availability

The data that support the findings of this study are available from the demographic and health survey database (https://dhsprogram.com/) after registration.
