# Supplementary material for: Empowering women can improve child dietary diversity in Ethiopia
Source: Matern Child Nutr. 2021 Nov 4;20(Suppl 5):e13285. doi: 10.1111/mcn.13285 (PMC11258775; doi:10.1111/mcn.13285)
Supplement: Supplementary file 1 — Table S1: Description of variables included in the construction of the SWPER index Table S2: Trends and changes in means or percentage of child diet and child diet determinants among children aged 6–23 months in Ethiopia for 2005, 2011, and 2016. Table S3 Change in SWPER index between 2005 and 16, by region [file MCN-20-e13285-s001.docx]

**Supplementary file**

Table S1: Description of variables included in the construction of the SWPER index

| **DHS questions** | **Code** |
| --- | --- |
| **Domain 1: Attitude towards Violence** | |
| Beating justified if wife goes out without telling husband | Justified= -1; don’t know=0; not justified =1 |
| Beating justified if wife neglects the children | Justified= -1; don’t know=0; not justified =1 |
| Beating justified if wife argues with husband | Justified= -1; don’t know=0; not justified =1 |
| Beating justified if wife refuses to have sex with husband | Justified= -1; don’t know=0; not justified =1 |
| Beating justified if wife burns the food | Justified= -1; don’t know=0; not justified =1 |
| **Domain 2: Social independence** | |
| Frequency of reading newspaper or magazine | Not at all=0; <once a week=1; ≥once a week=2 |
| Respondent worked in last 12 months | No=0; in the past year=1; have a job, but on leave past 7 days=2; currently working=2 |
| Woman’s education | Years |
| Education difference: woman’s minus husband’s years of Schooling | Years |
| Age difference: woman’s minus husband’s age | Years |
| Age at first cohabitation | Years |
| Age of respondent at 1st birth | Years |
| **Domain 3: Decision making** | |
| Who usually decides on respondent's health care | Husband or other alone= –1; joint=0; respondent alone=1 |
| Who usually decides on large household purchases | Husband or other alone= –1; joint=0; respondent alone=1 |
| Who usually decides on visits to family or relatives | Husband or other alone= –1; joint=0; respondent alone=1 |

The last step in the calculation of the individual scores is their standardization. We used the SWPER mean and standard deviations for the African region (zero value represents the average empowerment level of the Sub-Saharan African region). To standardize the SWPER score the following equations were used, and mean and standard deviations reported in Ewerling et al (1):

𝑆𝑡𝑑 𝑆𝑐𝑜𝑟𝑒 _𝐴𝑡𝑡𝑖𝑡𝑢𝑑𝑒 𝑡𝑜 𝑣𝑖𝑜𝑙𝑒𝑛𝑐𝑒𝑖_ = [(𝑆𝑐𝑜𝑟𝑒 𝐴𝑡𝑡𝑖𝑡𝑢𝑑𝑒 𝑡𝑜 𝑣𝑖𝑜𝑙𝑒𝑛𝑐𝑒_𝑖_) −(𝑚𝑒𝑎𝑛)] /𝑠𝑡𝑎𝑛𝑑𝑎𝑟𝑑 𝑑𝑒𝑣𝑖𝑎𝑡𝑖𝑜𝑛 ….(eq 1)

𝑆𝑡𝑑 𝑆𝑐𝑜𝑟𝑒 _𝑆𝑜𝑐𝑖𝑎𝑙 𝑖𝑛𝑑𝑒𝑝𝑒𝑛𝑑𝑒𝑛𝑐𝑒𝑖_ = [(𝑆𝑐𝑜𝑟𝑒 𝑆𝑜𝑐𝑖𝑎𝑙 𝑖𝑛𝑑𝑒𝑝𝑒𝑛𝑑𝑒𝑛𝑐𝑒_𝑖_) −(𝑚𝑒𝑎𝑛)]/ 𝑠𝑡𝑎𝑛𝑑𝑎𝑟𝑑 𝑑𝑒𝑣𝑖𝑎𝑡𝑖𝑜𝑛 …(eq 2)

𝑆𝑡𝑑 𝑆𝑐𝑜𝑟𝑒 _𝐷𝑒𝑐𝑖𝑠𝑖𝑜𝑛−𝑚𝑎𝑘𝑖𝑛𝑔𝑖_ = [(𝑆𝑐𝑜𝑟𝑒 𝐷𝑒𝑐𝑖𝑠𝑖𝑜𝑛−𝑚𝑎𝑘𝑖𝑛𝑔_𝑖_) − (𝑚𝑒𝑎𝑛)]/𝑠𝑡𝑎𝑛𝑑𝑎𝑟𝑑 𝑑𝑒𝑣𝑖𝑎𝑡𝑖𝑜n…………(eq 3)

**Table S2: Trends and changes in means or percentage of child diet and child diet determinants among children aged 6–23 months in Ethiopia for 2005, 2011, and 2016.**

| **Variables** | **2005** | **2011** | **2016** | **Change (2016-2005)** | **P-value** |
| --- | --- | --- | --- | --- | --- |
|  | **mean or %** | | | |  |
| Number of food groups a child ate (out 8) | 2.73 | 2.54 | 2.77 | 0.04 | 0.494 |
| MDD: 5 out of 8 food groups (%) | 5.36 | 4.62 | 12.57 | 7.21 | <0.001 |
| SWPER: Attitude to violence | -1.01 | -0.75 | -0.58 | 0.43 | <0.001 |
| SWPER: Autonomy/Social independence | -0.51 | -0.44 | -0.35 | 0.16 | <0.001 |
| SWPER: Decision making SWPER score | -0.11 | 0.06 | 0.26 | 0.37 | <0.001 |
| Wealth score (0-10) | 1.31 | 1.94 | 2.24 | 0.94 | <0.001 |
| Residence: Urban (%) | 7.24 | 12.99 | 11.92 | 4.68 | <0.001 |
| Respondent's current age in years | 29.08 | 28.27 | 28.73 | -0.36 | 0.288 |
| Attended 4+ ANC visits (%) | 13.49 | 17.25 | 34.29 | 20.80 | <0.001 |
| Number of children 5 and under | 1.89 | 1.84 | 1.76 | -0.13 | <0.001 |
| Child age in Months | 13.67 | 13.77 | 13.81 | 0.14 | 0.55 |

**Table S3 Change in SWPER index between 2005-16, by region**

| Characteristics | SWPER score: Attitude to violence | | | SWPER score: Autonomy/Social independence | | | SWPER score: Decision making | | |
| --- | --- | --- | --- | --- | --- | --- | --- | --- | --- |
|  | 2005 | 2011 | 2016 | 2005 | 2011 | 2016 | 2005 | 2011 | 2016 |
| **Region** |  |  |  |  |  |  |  |  |  |
| Tigray | -0.686 | -0.484 | -0.568 | -0.669 | -0.456 | -0.334 | -0.099 | 0.25 | 0.375 |
| Afar | -0.779 | -0.884 | -0.907 | -0.47 | -0.519 | -0.553 | -0.08 | 0.011 | 0.015 |
| Amhara | -1.139 | -0.72 | -0.529 | -0.834 | -0.753 | -0.529 | 0.101 | 0.143 | 0.331 |
| Oromia | -0.986 | -0.731 | -0.669 | -0.516 | -0.434 | -0.438 | -0.045 | 0.075 | 0.179 |
| Somali | -1.211 | -1.016 | -0.099 | -0.402 | -0.423 | -0.459 | -0.353 | -0.238 | 0.357 |
| Benishangul | -0.742 | -0.381 | -0.369 | -0.695 | -0.647 | -0.49 | -0.165 | -0.042 | 0.301 |
| SNNPR | -1.221 | -0.987 | -0.596 | -0.408 | -0.301 | -0.287 | -0.158 | 0.002 | 0.164 |
| Gambela | -0.491 | -0.518 | -0.287 | -0.649 | -0.458 | -0.22 | -0.059 | 0.113 | 0.462 |
| Harari | -0.599 | -0.679 | -0.175 | -0.274 | -0.187 | -0.215 | 0.508 | 0.385 | 0.449 |
| Addis Adaba | 0.236 | 0.453 | 0.583 | 0.812 | 0.484 | 0.976 | 0.773 | 0.765 | 0.776 |
| Dire Dawa | -0.39 | -0.392 | -0.145 | -0.282 | -0.083 | -0.157 | 0.435 | 0.221 | 0.445 |
